# Natural Chiral Scaffolds in Alzheimer′s Disease: Therapeutic Potential, Mechanism, and Clinical Aspects

**DOI:** 10.1155/bmri/5968529

**Published:** 2026-04-07

**Authors:** Aniket Singh, Sanjeev Kumar Sahu, Anil Kumar Sah

**Affiliations:** ^1^ School of Pharmaceutical Sciences, Lovely Professional University, Phagwara, Punjab, India, lpu.in; ^2^ Division of Quality Control, Gorkha Ayurved Company Pvt. Ltd., Gorkha Haramtari, Kathmandu, Nepal

**Keywords:** Alzheimer′s disease, chiral compounds, clinical aspects, natural product, phytoconstituent, therapeutic

## Abstract

Alzheimer′s disease (AD) is a multifactorial neurodegenerative disorder characterized by progressive cognitive impairment, amyloid‐*β* deposition, tau pathology, oxidative stress, neuroinflammation, and dysfunction of synapses. Current therapeutic options are largely symptomatic and lack disease‐modifying efficacy. Target binding, pharmacokinetics, and therapeutic efficacy in AD are all significantly impacted by the stereochemistry of many bioactive natural scaffolds, which are enantiomerically defined molecules. Due to the complexity of their stereochemical structures and their multiple‐target pharmacological attributes, natural chiral scaffolds have received significant attention as lead compounds to treat AD. The chirality has a critical impact on target selectivity, receptor binding, blood–brain barrier permeability, and pharmacokinetic behavior. By combining stereochemistry with pharmacological and clinical data, it is possible to expedite the discovery of safer and more effective disease‐modifying therapies, thus making chiral natural products attractive for AD drug discovery in the future.

## 1. Introduction

Memory loss, cognitive decline, and ultimately, difficulty with everyday life tasks are the hallmarks of Alzheimer′s disease (AD), a progressive neurological illness that primarily affects the elderly [1]. The number of people with AD and other dementias worldwide has more than doubled, from 21.8 million in 1990 to 56.9 million in 2021, as a result of both demographic shifts and higher risk per person [2]. The cause of AD could include long‐term oxidative damage, which causes neurons′ mitochondria to malfunction, thereby increasing the formation of reactive oxygen species (ROS) that harm proteins, lipids, and DNA. This exacerbates tau pathology and amyloid buildup. Hyperphosphorylated tau creates neurofibrillary tangles, and aberrant amyloid‐beta (A*β*) accumulation generates plaques, impairing neuronal transmission and leading to progressive neurodegeneration [3].

Current FDA‐approved treatments, such as cholinesterase inhibitors (donepezil, rivastigmine, and galantamine) and N‐methyl‐D‐aspartate (NMDA) receptor antagonists (memantine), merely reduce symptoms [4]. A wealth of bioactive chemicals with a variety of chemical structures and pharmacological characteristics can be found in natural products, which are obtained from plants, marine organisms, microorganisms, and other sources [5]. For multifactorial diseases like AD, which involve a network of pathological processes such as A*β* accumulation, tau hyperphosphorylation, oxidative stress (OS), neuroinflammation, mitochondrial dysfunction, and impaired autophagy, their multitarget potential makes them especially attractive [6]. In fact, a number of natural substances have demonstrated encouraging preclinical efficacy. Certain flavonoids, alkaloids, terpenoids, and polyphenols, for instance, have been shown to limit aberrant tau protein expression, modify cholinergic neurotransmission, decrease oxidative damage, and interfere with A*β* formation or aggregation [7]. A sophisticated approach to Alzheimer′s pathology is provided by the deliberate integration of multitarget‐directed ligands (MTDLs) with particular chiral linkages. According to recent research, the key to the effectiveness of chalcone‐based scaffolds, flavonoid derivatives, and sulfonylhydrazones is their stereoselective binding, which allows for precise interactions within the 20 Å‐deep AChE/BChE gorges to inhibit targets like carbonic anhydrase and BACE‐1. Although these chiral leads exhibit favorable CAS/PAS binding and submicromolar potency, their clinical transition depends on maximizing blood–brain barrier (BBB) penetration using cutting‐edge nanocarrier formulation techniques [8–10]. *Ginkgo biloba* (GB) is one of the natural extracts that has advanced into clinical trials and demonstrated mild yet noteworthy impacts on AD patients′ cognitive function [1]. However, problems like bioavailability, BBB penetration, toxicity, extract composition variability, and inadequate clinical validation make it difficult to turn natural product leads into approved anti‐AD medications [4]. Additionally, many natural anti‐Alzheimer compounds are chiral molecules whose stereochemistry strongly influences their pharmacological activity. Therefore, chiral chromatography becomes essential for identifying active enantiomers, ensuring quality control, and understanding structure–activity relationships (SARs) in herbal therapeutics.

This review is aimed at critically assessing the preclinical and clinical efficacy of important natural products in the context of AD pathogenesis, systematically examining their multitarget mechanisms, and discussing the main pharmacokinetic issues and future tactics needed for their successful clinical translation.

## 2. About AD

### 2.1. Epidemiology

Estimates suggest that in 2019, about 51.6 million people all over the world had AD and other dementias. This equals around 683 cases for every 100,000 people. Between 1990 and 2019, the global rate grew by a moderate 5.7%. However, regional and national changes differed significantly: China (+29.2%) and Japan (+22.3%) showed the largest national increases, whereas East Asia and the high‐income Asia Pacific region saw the largest gains, rising by 28.3% and 19%, respectively. In contrast, Western Sub‐Saharan Africa showed a decrease of 2.4%. The most substantial national declines were seen in high‐income nations such as Spain (13%) and Luxembourg (10.7%) [11].

### 2.2. Pathogenesis

**Figure 1:**
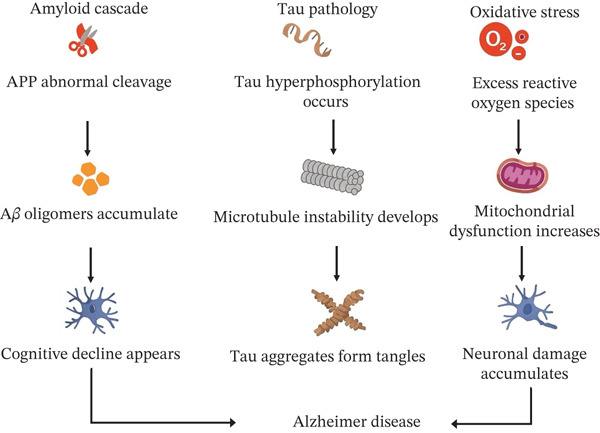
Pathology of Alzheimer′s disease.

The onset of Alzheimer′s is linked to the incorrect cleavage of amyloid precursor protein (APP) by *β*‐ and *γ*‐secretases, which results in the production of A*β* peptides. Accumulation of certain A*β* forms, notably A*β*42, begins many years before the emergence of any clinical symptoms [12]. A*β* peptides can produce hazardous soluble oligomers that can pose risks by disturbing neuron communication, impacting synaptic plasticity, and causing dendritic spine loss [13]. At the same time, the microtubule protein tau gets phosphorylated incorrectly. It separates from microtubules and clumps together to form paired helical filaments and neurofibrillary tangles. The amount and spread of tau pathology are linked to cognitive decline [14]. Tau and A*β* interact in feed‐forward loops, where pathogenic tau increases synaptic dysfunction and neuronal death while A*β* oligomers drive tau phosphorylation and mislocalization [15]. These protein changes cause brain immune cells (microglia and astrocytes) to become persistently activated, releasing inflammatory mediators that worsen neuronal damage and prune synapses. Neuroinflammation results from this, aggravating A*β*/tau pathology [16]. Simultaneously, neurons suffer from increased OS and mitochondrial dysfunction: energy production declines and ROS deteriorate proteins, lipids, and DNA, accelerating synaptic failure and cell death [17]. Vascular changes and a breakdown of the BBB can reduce waste removal (including A*β*) and change how nutrients and oxygen are delivered, which can lead to poorer brain health [18]. AD arises from several combined mechanisms. These mechanisms involve A*β* oligomers, tau protein aggregates, long‐term brain inflammation, OS coupled with mitochondrial problems, poor waste removal, and blood vessel issues. These problems can cause a gradual loss of synapses, disrupted brain networks, and nerve cell death, leading to memory loss and loss of function [19]. All these molecular pathways are summarized as given below (Figure 1)

#### 2.2.1. Amyloid Cascade

The amyloid cascade hypothesis suggests that the buildup of amyloid‐2 (A2) peptides, derived from APP, represents the initial disease‐causing step in AD [20]. APP undergoes unusual processing through *β*‐ and *γ*‐secretase pathways, which leads to the production of longer A*β* variants, especially A*β*42, that are prone to aggregation [21]. The subsequent formation of extracellular amyloid plaques starts a series of events. This leads to tau hyperphosphorylation, neurofibrillary tangles, synaptic dysfunction, nerve cell death, and eventually, dementia [20]. Plaque buildup leads to microglial activation, which causes the synthesis of proinflammatory cytokines and neuroinflammation through complement activity. These processes hasten the decline of neuronal networks [22]. Research indicates that mutations in the APP or PSEN1/2 genes cause the production of A*β*, which is associated with early‐onset familial AD [23]. These mutations usually increase the A*β*42/A*β*40 ratio, which raises aggregation and causes early and severe disease [24]. A*β* oligomers are thought to be the most harmful form of A*β*. They disrupt calcium balance, hurt mitochondrial function, cause OS and inflammation, and lead to synaptic loss. These harmful activities add to nerve damage and cognitive decline in AD [25].

#### 2.2.2. Tau Protein

Tau, a protein linked to microtubules, helps keep neuronal microtubules stable. It exists in neurons where it stabilizes and crosslinks axonal microtubules to make bundles. Problems with tau regulation cause Alzheimer′s and other neurodegenerative diseases called tauopathies [26]. In Alzheimer′s, unusual changes after protein production, especially excessive phosphorylation, make tau separate from microtubules and build up as neurofibrillary tangles, leading to nerve damage [27]. Tau hyperphosphorylation encourages microtubule instability and neuronal problems. It is linked to the neuropathological signs of AD, like extracellular deposits and intracellular neuronal clumps. Several kinases, like CDK5, GSK3*β*, and ERK2, have been related to strange tau phosphorylation. These kinases are important in how *β*‐APP is processed, as well as the neurotoxicity it causes [28]. Tau clusters disturb microtubule construction, hinder axonal material movement, impair mitochondria, and cause synapse and neuron loss. These disturbances lead to neurodegeneration in tau diseases [29]. Animal studies show that too many human Tau proteins harm cognitive ability, trigger inflammasome activation and microglia activation, and promote disease progression in mice [30]. Tau pathology shows a greater correlation with cognitive decline and neurodegeneration compared to amyloid plaque burden. This suggests a key part it plays in how the disease develops [31].

#### 2.2.3. OS

An imbalance between ROS and the cell′s antioxidant defenses is known as OS [32]. The brain′s high oxygen consumption and high polyunsaturated fatty acid content make it especially susceptible to ROS/RNS damage [33]. According to certain research, OS is an early event in pathogenesis since OS occurs before tau hyperphosphorylation and A*β* plaque development [34]. OS directly leads to the pathogenesis of AD by chemically altering A2, such as the oxidation of methionine‐35, which increases its dimerization and oligomerization, and thus, its neurotoxicity [35]. Additionally, it alters the redox balance in neurons, resulting in the downregulation of phosphatases like PP2A and the upregulation of tau‐phosphorylating kinases like GSK‐3*β*. This leads to tau hyperphosphorylation and the development of neurofibrillary tangles [36]. OS also damages mitochondria, which leads to decreased energy production and an excess of ROS, which in turn speeds up neuronal damage in a vicious cycle [37]. Furthermore, OS produces AGEs that attach to RAGE receptors, initiating inflammatory pathways that involve TNF‐*α* and IL‐1, thereby accelerating tau and A*β* pathology and stimulating chronic neuroinflammation. When combined, these processes not only contribute to the fundamental disease process but also greatly quicken AD′s development [38].

## 3. Conventional Treatment for AD

Currently, no treatment can totally stop or reverse AD. The main goals of conventional treatment are to improve quality of life, decrease the course of the disease, and relieve symptoms. The majority of approved medications work by either blocking pathological processes like amyloid buildup or altering neurotransmitter networks.

### 3.1. Cholinesterase Inhibitors (Acetylcholinesterases [AChEIs])

Acetylcholine (ACh) is the brain′s primary chemical transmitter for cognitive function. A decrease in ACh levels has been identified as a common cause of AD. AChE enzymes are responsible for hydrolyzing ACh neurotransmitters [39, 40]. Thus, one possible treatment approach is to raise the brain′s cholinergic levels by blocking AChE′s biological activity. As a result, AChE inhibitors are utilized to prevent AChE from degrading. By raising the concentration of ACh, AChE inhibitors can improve brain cell activity [41].

Cholinesterase inhibitors (AChEs) represent a frequently prescribed class of medications for managing cognitive symptoms tied to AD. They function by addressing a core neurochemical issue: reduced levels of ACh, a neurotransmitter. In AD, the breakdown of cholinergic neurons within the basal forebrain and hippocampus leads to a noticeable drop in ACh. This, in turn, impacts synaptic transmission, which is important for memory, learning, and attention [42]. AChEIs work by stopping the AChE enzyme from breaking down ACh in the synaptic cleft. This boosts ACh′s effects and betters cholinergic neurotransmission [43]. By inhibiting this enzyme, these medications help maintain larger amounts of ACh at neuronal synapses, somewhat compensating for cholinergic neuron loss reported in Alzheimer′s patients [44].

Among the FDA‐approved AChEIs, donepezil, rivastigmine, and galantamine are the most extensively used medicines, each differing in pharmacological profile, metabolism, and side effect spectrum [45].

Donepezil, a drug that selectively stops an enzyme in the brain from breaking down a key neurotransmitter, can help people with AD think more clearly and manage daily life better. It works for those with mild to severe forms of the illness [46]. Studies suggest that donepezil can help people with AD (mild, moderate, or severe) if they take it for 12–24 weeks. People who took donepezil showed some improvement in thinking, daily activities, and general condition. A dose of 10 mg/day worked a bit better than 5 mg/day. But a dose of 23 mg/day did not work any better and caused more side effects, so it is not a good option [47].

Rivastigmine, a pseudo‐irreversible agonist of both butyrylcholinesterase (BChE) and AChE, blocks these enzymes in two ways. Studies suggest it can enhance thinking and behavior in patients, especially those suffering from dementia related to Parkinson′s disease [48]. Rivastigmine, a cholinesterase inhibitor, is useful in treating AD, with stronger doses leading to better results. The 9.5‐mg/24‐h skin patch slowly releases the drug, avoiding blood level changes that could cause problems for the patient. Rivastigmine′s balance of water solubility, fat solubility, and small molecular size (C_44_H_22_N_2_O_2_) makes it suited for skin patches. This small patch (3.5 cm diameter, 10 cm^2^ area) causes nausea and vomiting less often (three times less), but it still works as well as the highest capsule doses. Because it sticks well, is tolerated by patients and provides easy, high‐dose treatment, it is a step forward in Alzheimer′s care [49].

Galantamine improves cholinergic signals in ways outside just blocking enzymes. It acts as an allosteric modulator on nicotinic ACh receptors and also works as a reversible competitive inhibitor of AChE [50].

Clinical data from 10 randomized, double‐blind, placebo‐controlled studies conducted across 6 months show that donepezil, galantamine, and rivastigmine resulted in a small cognitive boost (average ADAS‐Cog −2.37 points; *p* < 0.00001) for patients with mild to severe Alzheimer′s. Some benefits were noted in behavior, daily activities, and general condition. However, cholinesterase inhibitors caused more side effects, mainly nausea, vomiting, and diarrhea, which led to more patients stopping treatment (29% compared with 18% with placebo). Two studies hinted that similar positive changes occurred even in severe dementia. Donepezil had fewer negative impacts, but a 2‐year study comparing it with rivastigmine showed no changes in cognitive ability or how well patients could function. In general, AChEIs give short‐term relief from symptoms but do not stop the illness from getting worse or prevent nerve damage [51].

### 3.2. NMDA Receptor

NMDA receptor antagonists represent a key class of drugs for the treatment of moderate‐to‐severe Alzheimer′s. They mainly work by protecting brain cells from problems with glutamate signaling [52]. In AD, too much activity of NMDA receptors leads to a large amount of calcium influx into neurons. This causes cell damage and neuron death, which leads to cognitive problems [53]. Memantine is the only NMDA receptor antagonist approved for treating Alzheimer′s [54]. It works by blocking NMDA receptors that are abnormally active, and it binds only when they are overstimulated [55]. This targeted blocking helps steady glutamate activity in the brain, preventing extra neuron damage but still allowing normal brain signals to pass through [56].

Memantine is often prescribed at 20 mg a day (10 mg twice a day) or as a 28‐mg extended‐release tablet taken once a day [57]. Studies show memantine helps with thinking, everyday tasks, and general well‐being, especially for those with moderate‐to‐severe AD [54, 58]. One study lasting 24 weeks with about 400 participants found that memantine led to better results in cognitive and functional tests when compared with a placebo [59].

Memantine, although not curing the underlying neurodegeneration, can help manage symptoms and slow functional decline in later stage Alzheimer′s. This often improves the quality of life for both patients and their caregivers [54].

### 3.3. A*β* Targeting Monoclonal Antibodies

Current progress has turned attention to treatments that change the course of the disease by focusing on A*β* plaques, a key feature of Alzheimer′s. Two drugs approved by the FDA, aducanumab and lecanemab, represent the first treatments created to change the basic disease process, instead of just easing symptoms. [60].

Aducanumab is a human monoclonal antibody that selectively binds aggregated A*β*, promoting its clearance from the brain via microglial phagocytosis. It was granted accelerated FDA approval in 2021 for early‐stage AD [61]. Aducanumab went through two big studies, EMERGE and ENGAGE. EMERGE (with 1638 patients) showed that a high dose slowed down the disease by about 22% and lowered amyloid levels in the brain, as seen on PET scans. ENGAGE (with 1647 patients) did not find much clinical improvement, but it did show amyloid reduction, probably as a result of not enough people getting the highest dose for long enough. EMERGE suggests aducanumab might slow Alzheimer′s, whereas ENGAGE shows it can clear amyloid without a clear clinical benefit. This leads to ongoing debate about how well it works [62, 63].

Lecanemab (Leqembi) is an anti‐A*β* monoclonal antibody that targets soluble A*β* protofibrils to reduce amyloid buildup and prevent cognitive deterioration. It got full FDA approval in 2023 based on the CLARITY‐AD Phase III trial, which involved 1795 people with early Alzheimer′s [60]. Lecanemab, after 18 months of treatment, slowed clinical decline by 27% versus placebo, as measured by the CDR‐SB scale. PET scans showed a measurable decrease in amyloid buildup. Patients getting treatment also had better scores on the ADAS‐Cog14 and ADCS‐MCI‐ADL scales, suggesting cognitive and functional improvements [60]. Monoclonal antibodies can cause amyloid‐related imaging abnormalities, like edema or microhemorrhages. Because of this, MRI monitoring is needed during treatment. [64].

## 4. Herbal Therapeutics in AD

AD is a neurodegenerative disease that causes memory loss and cognitive decline due to tau hyperphosphorylation, OS, A*β* accumulation, and neuroinflammation [65]. Only symptomatic relief is provided by currently approved drugs like donepezil, rivastigmine, galantamine, and memantine, which have limited effectiveness and obvious side effects like nausea, bradycardia, and dizziness [66].

Due to their multitarget mechanisms, which include anti‐inflammatory, antiamyloid, and antioxidant effects, herbal remedies have recently become intriguing options for the treatment of AD. In mild‐to‐moderate dementia, *Ginkgo biloba* extracts (GBEs) (EGb 761) have shown moderate but significant improvements in cognition and everyday functioning when compared with a placebo; in certain trials, the results have been comparable with those of low‐dose donepezil [67]. Similar to this, huperzine A (HupA), an alkaloid derived from *Huperzia serrata*, acts as a reversible AChE inhibitor and NMDA receptor antagonist, demonstrating notable cognitive benefits in several randomized controlled trials [68]. Research suggests saffron may work as well as donepezil and memantine to improve behavior and thinking, and it seems to cause fewer side effects. [69]. Curcumin, a compound in *Curcuma longa*, shows protective qualities for the brain, reduces amyloid plaques, and lessens inflammation in lab tests. Current studies with humans suggest that specially made versions of curcumin that the body can use may help improve memory and focus in people without dementia [70]. Traditional Chinese medicine makes use of multiherb formulas. These appear to offer extra help for thinking and behavior, mostly when used with standard treatments [71].

Herbal remedies, when contrasted with traditional drugs, offer multitarget regulation of AD development. They show fewer cholinergic side effects, better tolerance, and possible extra benefits [72]. Herbs and herbal medicines have been used traditionally for a long time and seem safe and work well, but more research is needed [73]. Herbal treatments, such as GB, HupA, saffron, and curcumin, can be helpful additions to Alzheimer′s patient care. When doctors oversee their usage, these supplements might lead to better thinking skills and a better quality of life [74].

### 4.1. Role of Chirality in Treatment of AD

AChE and BChE are serine hydrolases. They control cholinergic neurotransmission by breaking down ACh. They have a ~20 Å active‐site gorge with a catalytic/acylation site (A‐site) at the bottom and a peripheral site (P‐site) near the opening. In AChE, the A‐site has the catalytic triad Ser‐His‐Glu and a choline‐ and acyl‐binding pocket made up of Trp86, Tyr337, Phe295, Phe297, and Trp236. The P‐site is full of aromatic residues (Tyr70, Tyr121, Trp279/Trp286, Tyr124, Tyr72, Tyr341, Tyr334, and Asp72) that direct ligand placement near the catalytic center. On the other hand, BChE has fewer aromatic residues in its gorge. It only keeps Tyr332 in the P‐site and Trp82, Phe329, and Trp231 in the A‐site, with Asp70 and Trp82 in the cysteine *Ω*‐loop connecting the two regions. Such structural variations change the gorge shape and ligand interactions, leading to different substrate and inhibitor binding in AChE and BChE. This allows chiral ligands to take on different positions and interactions within each enzyme. This leads to activity and selectivity that depends on stereochemistry [75, 76]. In AD, the cholinergic enzymes AChE and BChE function as pathological chaperones that speed up the creation of harmful protein clusters. AChE speeds up early‐stage A*β* oligomerization through its Peripheral Anionic Site (PAS). At the PAS, the hydrophobic channel interacts with A*β* peptides, creating very toxic insoluble fibrils. As the disease progresses and AChE activity decreases, BChE aids the malignant maturation of these structures. This change occurs at BChE′s activation site (Residues A277–Y282) near the opening of its catalytic gorge and through its carboxy terminus, which steadies soluble A*β* into complex plaques. The importance of these sites is supported by 5XFAD/BChE‐KO mouse models, where the lack of the BChE gene causes a large drop in plaque density, and by data showing that normal levels of BChE greatly accelerate fibrillization when compared to cognitively normal samples [77, 78]. Galantamine inhibits AChE (IC_50_ = 4.0 *μ*M) about twice as much as it inhibits BChE. Its shape lets it fit into the AChE active site, interacting with specific amino acids (Phe288, Phe290, and Trp84). Key interactions include hydrogen bonds with Glu199, Asp72 (or Y337), and Ser200. It also affects nicotinic acetylcholine receptors (nAChRs) and binds to A*β*(1–40), which stops oligomers from forming [79].

**Figure 2:**
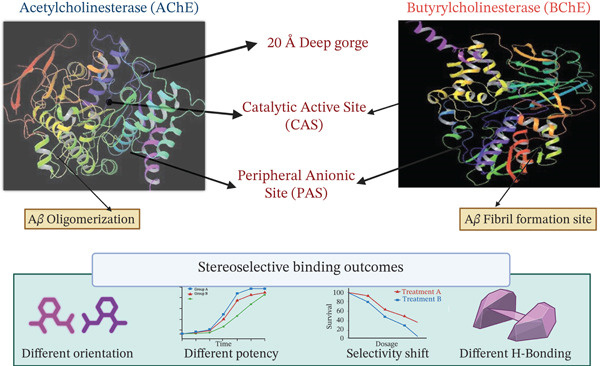
Chirality‐dependent modulation of AChE and BChe in Alzheimer′s disease.

Naturally sourced chiral compounds are still very helpful in treating AD because they have different structures and stereochemical traits that make them more biologically active (Figure 2). Galantamine and HupA, which are used in clinics, show how natural chiral alkaloids can change cholinergic function in AD. Also, some natural polyphenols and other chiral bioactives, like epigallocatechin gallate (EGCG), quercetin, and resveratrol, are being studied in clinical trials to see if they can protect the brain and improve thinking. Almost 100 natural molecules have been tested in preclinical and clinical research, and many have stereochemistry‐related ways of working that fight nerve damage and help improve brain function. These results suggest that chiral natural products could be useful for treating AD. Further clinical research is needed to completely understand how these products can help in making AD drugs [80]. Ginkgolides and bilobalide, which are terpenoids from GB and possess chirality, may help improve cerebral blood flow, lower OS, and stop A*β* aggregation. The primary active element was found to be (−) galantamine, which impacts the cholinergic system through a dual mechanism [72] [81]. EGCG, a catechin from green tea that is chiral, shows antioxidant and anti‐amyloid traits. Its natural (−) enantiomer seems more biologically active than its mirror image [82, 83]. Stereochemistry is very important in the drug industry because it changes how well drugs work. Sorting chiral molecules is key to understanding how chiral drugs act. Usually, one form of the molecule aids, while the other is ineffective or causes problems. Therefore, being able to tell chiral molecules apart is especially vital when making good drugs for Alzheimer′s [84].

### 4.2. Natural Product With Chiral Phytoconstituents for AD

Natural products rich in chiral phytoconstituents have become promising therapeutic candidates in the management of AD because of their structural and stereoselective biological activities. Chirality plays a crucial role in determining pharmacodynamics and pharmacokinetics, whereby enantiomers often show a significant difference in target binding, efficacy, and safety. The receptor selectivity and BBB permeability are stereochemical effects of these phytoconstituents, which increase therapeutic precision. Their bioavailability and clinical potential have also been enhanced by the developments in chiral separation, stereochemical characterization and a nanocarrier‐based delivery system. Overall, chiral phytoconstituent of natural products is a highly promising yet unexploited source of safer, multitargeted intervention against AD (Table 1).

**Table 1:** Natural products used for treatment of AD.

Phytoconstituents with source	Mechanism of action	Key clinical evidence	Comparative benefit to conventional drugs	Safety/notes	Chirality/desired enantiomer	Ref.
Galantamine (*Galanthus* spp. [snowdrop])	Acetylcholinesterase inhibitor; allosteric modulator of nicotinic receptors	FDA‐approved; improves cognition in mild–moderate AD	Comparable efficacy with donepezil/rivastigmine; fewer GI side effects	Generally well‐tolerated; mild GI symptoms possible	(−)‐Galantamine	[85] [81]
Huperzine A (*Huperzia serrata*)	Acetylcholinesterase inhibitor	Clinical trials show cognitive improvement	Similar efficacy to galantamine; possibly better tolerability	Mild side effects (GI, insomnia); long‐term safety less established	2R,9R,10R,11R natural stereoisomer	[86] [87]
Curcumin (*Curcuma longa* [turmeric])	Antioxidant, anti‐inflammatory, inhibits amyloid/tau aggregation	Mixed clinical results; some cognitive benefit	Multitarget; less effective than AChE inhibitors in trials (refrence)	Safe at dietary doses; poor bioavailability; high doses may cause GI upset	No chirality.	[88] [89]
Resveratrol (Grapes, *Polygonum*)	Antioxidant, anti‐inflammatory, modulates amyloid/tau	Early trials: Improved biomarkers, limited cognitive effect	Adjunctive benefit; not superior to AChE inhibitors	Well‐tolerated; high doses may cause GI symptoms	Trans‐resveratrol	[90] [91] [92]
Quercetin (Fruits/vegetables)	Antioxidant, anti‐inflammatory, inhibits amyloid aggregation	Preclinical and limited clinical evidence	Multitarget; less potent than standard drugs	Safe in food; high doses may cause kidney issues	No enantiomers	[93]
Saffron (*Crocus sativus*)	Antioxidant, antiamyloid, anti‐inflammatory	RCTs show comparable efficacy with donepezil/memantine in mild–moderate AD	Similar cognitive improvement, fewer adverse events	Generally well‐tolerated	No enantiomers	[69]
Epigallocatechin gallate (EGCG) (Green tea)	Antioxidant, antiamyloid, anti‐inflammatory	Animal and early human studies show cognitive benefit	Synergistic with other agents; not superior alone	Safe in moderate doses; high doses may cause liver toxicity	(−)‐EGCG	[83] [94]
Chinese herbal formulas (e.g., Danggui Shaoyao San)	Multitarget (A*β* clearance, antioxidant, neurotrophic)	Meta‐analyses show cognitive and behavioral improvement as adjunct therapy	Synergistic effects with conventional drugs	Variable formulations, need standardization	N/A	[71]
Ginsenosides (*Panax ginseng*)	Inhibition of A*β* production and accumulation, tau hyperphosphorylation, inhibition of oxidative stress, neuroinflammation	Some clinical trials show cognitive improvement	Adjunctive benefit	Well‐tolerated; insomnia and GI upset possible	Rb1 and Rg1	[95] [96] [97]
Diosgenin (*Dioscorea batatas* [yam])	Antiamyloid, neuroprotective	Preclinical evidence; limited human data	Not directly compared	Safe in food; supplement safety not fully established	Used as whole	[98] [99]
*Ginkgo biloba* extract (*Ginkgo biloba*)	Antioxidant, anti‐inflammatory, improves blood flow, modulates neurotransmitters	Multiple RCTs: Modest cognitive benefit, especially EGb 761	Comparable with AChE inhibitors in mild–moderate AD; fewer side effects	Generally safe; rare bleeding risk, mild GI symptoms	Natural fixed stereochemistry	[67]
*Withania somnifera* (Ashwagandha) (*Withania somnifera*)	Antioxidant, anti‐inflammatory, Neuroprotective, promotes synaptic regeneration	Preclinical and early clinical evidence	Not directly compared	Well‐tolerated; mild GI upset	Used as whole	[100]
Baicalein (*Scutellaria baicalensis*)	Antioxidant, anti‐inflammatory, inhibits amyloid/tau, neuroprotective	Preclinical evidence	Not directly compared	Safe in food; supplement safety not fully established	Not chiral	[101] [102] [103]
Tanshinone (*Salvia miltiorrhiza*)	Improve cognitive function, neuroprotective and neuropathology	Preclinical evidence	Not directly compared	Safe in food; supplement safety not fully established	Not available	[104] [105]
Berberine (*Berberis* spp.)	Neuroprotective, targeting amyloid beta plaques, neuroinflammation, and oxidative stress	Preclinical and limited clinical evidence	Not directly compared	GI side effects at high doses	Not available	[106]
Crocin/Crocetin	Restrained neuroinflammation, ameliorating the cognitive dysfunction	Preclinical Evidence	Not directly compared	No information available	All trans form	[107] [108]

#### 4.2.1. Ginkgolides

GBEs may help treat AD. GB, a gymnosperm from Japan, China, and Korea, is part of the Ginkgoaceae family and Ginkgoopsida class. It has many bioactive substances, like terpenoids (Ginkgolides A, B, and C), polyphenols, organic acids, and flavonoids (quercetin, kaempferol, and isorhamnetin). These substances have anti‐inflammatory, antioxidant, and antiapoptotic properties, making GB a popular traditional medicine for various health issues for many years [109]. GBE (EGB 761) is a well‐known supplement that older adults take to improve memory and slow down thinking decline. These properties may make it a viable treatment for AD [110].

GBE contains 6% terpenoids (3.1% Ginkgolides A, B, C, and J, and 2.9% bilobalide), as well as 24% flavonoid glycosides (which include quercetin). Its composition also features kaempferol, isorhamnetin, and 5%–10% organic acids. It is believed that flavonoids and terpenoids make up the active parts of GBE [111]. GBE, which includes active ingredients such as flavonoids and terpene lactones (found in antidementia drugs like EGb761 and GBE50), protects nerves. It does this by working as an antioxidant and preventing cell death. GBE gets rid of free radicals and stops cell death caused by issues with mitochondria. This treatment addresses important parts of AD by lowering OS from mitochondrial ROS, preventing A*β* clumping, fixing mitochondrial function, and improving blood flow in the brain [112].

Recent work suggests that GBE may protect nerve cells from damage caused by A*β* by interfering with glucose absorption, reducing ROS accumulation, blocking AKT activation, improving mitochondrial health, and suppressing JNK and ERK 1/2 signaling, as well as programmed cell death [113]. GBE can decrease A*β* creation in the brain. It does this by reducing free cholesterol levels, which may influence APP processing and amyloid formation [114].

#### 4.2.2. HupA

HupA is a sesquiterpene alkaloid extracted from the Chinese clubmoss *H. serrata*. The naturally occurring alkaloid HupA, which is derived from the Chinese clubmoss plant *H. serrata*, has drawn attention because it has several neuroprotective properties and can inhibit cholinesterase. HupA has a chiral center and exists as two forms: the natural levorotatory (−)‐HupA and the synthetic dextrorotatory (+)‐HupA. The research on HupA analogues suggests that the levorotatory forms are better at blocking AChE compared with the dextrorotatory forms. For this reason, (−)‐HupA is often chosen for treating AD [87]. Strong AChE inhibitory activity is one of the many pharmacological actions of HupA and its derivatives. HupA has neuroprotective properties that protect neurons from excitotoxicity and OS, potentially helping to preserve cognitive function [86]. The neuroprotective effects of HupA treatment originate from cholinergic signaling, which increases neurotrophic factor expression, inhibits the N‐methyl‐D‐aspartate receptor (NMDAR), regulates ROS, and promotes neuronal survival [115]. HupA is a potent, reversible, and selective inhibitor of AChE. After HupA is administered, ACh will start to accumulate since HupA inhibits AChE. ACh signaling mainly uses *α*7nAChRs and *α*4*β*2nAChRs to produce an anti‐inflammatory response [116]. According to a Phase II trial, HupA (200 *μ*g twice daily) is not effective in treating AD. Further research on the maximum tolerated dose and long‐term effects is necessary because secondary analysis indicates that a higher dose (400 *μ*g twice daily) may improve cognition [117]. In individuals with AD, HupA appears to have some positive effects on daily living activities, cognitive function, and overall clinical evaluation [118].

#### 4.2.3. Galantamine

Galantamine, an alkaloid found naturally, comes from the Amaryllidaceae family. In the 1950s, researchers in the Soviet Union first separated it from the Caucasian snowdrop and later from various Amaryllidaceae plants′ bulbs [119]. In nature, this compound appears as the (−)‐enantiomer, which is the form with pharmacological activity employed in clinical practice [81].

Galantamine acts in two ways. It inhibits AChE and also modulates nAChRs, the only AD treatment drug known to do both. This second action on nAChRs is key because the reduction of nAChRs contributes to diminished cholinergic neurotransmission in AD patients. Four randomized, double‐blind, placebo‐controlled studies were conducted. These studies lasted up to 6 months and showed that 16 and 24 mg/day of galantamine improved cognitive and global function. It also helped with daily living activities and behavior for up to 6 months when compared with placebo and at the start of the study. In 2001, following 50 years of study, the FDA approved (−)‐galantamine hydrobromide, sold as Razadyne, for treating mild‐to‐moderate dementia from AD. A review of studies on galantamine showed that it improved thinking, behavior, and overall condition in Alzheimer′s patients [120].

#### 4.2.4. Crocin

Research indicates that crocin, a yellow carotenoid and the main component of *Crocus sativus* L. extract, may have anti‐inflammatory, antidepressant, memory improvement, and antiapoptotic action [121]. Crocin is mostly found in the all‐trans form, but cis‐isomers are also present. Crocins can be grouped by how many and where *β*‐D‐glucopyranoside groups are on the crocetin structure, for example, trans‐crocin 4 and other similar compounds [122]. Crocin acts on important pathways like PI3K/AKT. It also lowers OS and controls proteins linked to apoptosis. Crocin can also help mitochondria work better and improve synaptic plasticity [107]. In 17 in vitro and in vivo preclinical studies, saffron has been demonstrated to alleviate cognitive impairment in AD animal models. Crocin seems to control glutamate levels, lower OS, and modify the aggregation of tau and A*β* proteins. Saffron has a better safety profile and comparable effects on cognitive impairment to donepezil and memantine, according to just four clinical studies [69].

#### 4.2.5. Emerging Chiral Scaffolds

**Figure 3:**
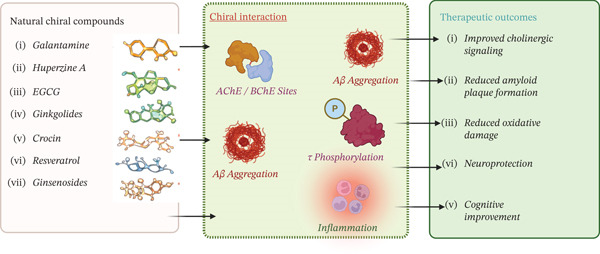
Chirality‐driven multitarget mechanisms of natural compounds in Alzheimer′s disease.

Recent studies support the information about chalcone derivatives as adaptable scaffolds for multitarget anti‐AD design, their micromolar AChE/BChE inhibitory activity, improved multitarget profiles when linked to pharmacophores like rivastigmine and morpholine, and molecular docking/SAR analyses rationalizing binding in enzyme gorge subsites (Figure 3) [127]. In addition to molecular docking that explains binding modes and stability in active sites, studies show that halogenated chalcone derivatives with Ki values in the low nanomolar range significantly inhibit AChE and BChE. Furthermore, chalcone hybrids that combine pharmacophores like rivastigmine exhibit increased potency and selectivity against AChE and BChE; molecular docking confirms important interactions, and SAR studies highlight substitution effects on activity. Nevertheless, the majority of active chalcones are still in the preclinical stage and face difficulties such as exposure to the central nervous system (CNS) [8, 124].

Sulfonylhydrazone and similar hydrazone derivatives can act as ligands that inhibit cholinesterases (AChE and BChE) and other enzymes relevant to AD, like human carbonic anhydrases, which relate to OS and mitochondrial problems. Several new sulfonylhydrazone structures show good AChE inhibition and dock well at catalytic and peripheral anionic locations (CAS/PAS), supporting a multitarget drug design approach. The hydrazone part allows quick SAR study, and chirality is important for their biological action. Chiral hydrazide‐hydrazone derivatives display different conformational isomers (E/Z), with the E‐E conformer being the most stable, which affects enzyme inhibition strength and binding. However, these compounds do not yet have in vivo CNS pharmacokinetic and safety information, which limits their progress toward clinical use. Molecular docking studies show consistent, strong binding between these chiral sulfonylhydrazones and AChE and carbonic anhydrase active sites, suggesting they could be good starting points for more work [9, 125, 126].

Since 2020, computer docking research has found that flavonoids like ginkgetin, kolaflavanone, epicatechin/epigallocatechin derivatives, luteolin, and similar flavones can strongly stop AChE, BChE, and other Alzheimer′s targets such as BACE‐1. These studies show good hydrogen bonding, *π*–*π* stacking, and hydrophobic actions with important parts of the enzyme active sites. They predict binding strengths from micromolar to submicromolar, which match experimental blockage data. The stereochemistry of flavonoid structures, involving chiral glycosides and conformationally biased flavonoids, greatly affects predicted binding shapes and strengths. This suggests that enantiomeric or atropisomeric forms should be tested to see how their biological actions differ. Even with good computer and lab results, flavonoids have problems with how well they are absorbed and how easily they cross the BBB. Current work is fixing these problems with better formulations and nanocarrier methods. In general, thinking about chirality when designing flavonoids with docking helps people understand how well they can block cholinesterases and AD‐related enzymes on different targets. [10, 127, 128]

Several problems exist in these scaffolds include limited exposure in the living CNS, not enough biological evaluation that is specific to enantiomers, and not enough data on long‐term safety. To move these leads toward clinical use, it is a good idea to routinely separate and characterize the enantiomers of lead compounds because chirality greatly changes how they move through the body and their toxicity. Early use of BBB penetration tests and pharmacokinetic studies in living organisms is needed to measure CNS exposure. Using multitarget biochemical and cell‐based profiling, along with biochemical tests, like AChE, BChE, and antiaggregation assays, can give detailed activity data. Also, formulation methods like prodrugs or lipid/nanocarrier systems are important to make bioavailability and BBB penetration better. Current reviews of methods stress how important enantioselective techniques are for correctly tracking how drugs move through the body and talk about improvements in chiral separation methods that are critical for these translational steps [129–132].

## 5. Clinical Aspects of Natural Products in the Treatment of AD

The various aspects of clinical study for natural products in the treatment of AD. Some clinical studies and key findings for GB and *H. serrata* were summarized in Tables 2 and 3. Although EGb 761 demonstrated modest cognitive improvement (SMD approximately −0.71 at 240 mg/day), long‐term prevention trials such as Guid Age failed to show a significant reduction in AD incidence (HR 0.84, *p* = 0.306), suggesting primarily symptomatic rather than disease‐modifying benefits [110].

**Table 2:** Key clinical findings and results of *Ginkgo biloba.*

Study type	Extract /dose	Population (*N*)	Duration	Cognitive outcomes	Quantitative results	Safety profile	Ref.
Systematic review of 15 clinical trials in AD and dementia.	Standardized *Ginkgo biloba* extract, mainly EGb 761.	Not specified	Treatment period 4–24 weeks.	11/15 trials showed improvement in cognitive function, neuropsychiatric symptoms, and functional abilities; four trials reported no significant difference versus placebo.	Significant differences reported for MMSE, SKT, and NPI in responsive trials.	The review emphasizes need for more thorough assessment of adverse effects, long‐term use, and interactions; tolerability is broadly acceptable but not exhaustively characterized.	[110]
Seven randomized controlled trials, *N* = 939 in total.	“*Ginkgo biloba* preparation” (mostly standardized extracts, but not restricted to EGb 761 by name).	Adults with Alzheimer′s disease (AD), diagnosed by standard criteria.	Trial durations varied (not all exactly reported here; most AD Ginkgo trials are ~22–26 weeks).	Significant benefits on cognitive function and global clinical assessment vs placebo.	Risk ratio for response in cognitive function 1.98 (95% CI 1.52–2.59, *Z* = 5.12, *p* < 0.001).CGIC odds ratio 3.119 (95% CI 2.206–4.410, *Z* = 6.44, *p* < 0.001)—rounding explains minor difference.	Adverse events were mild, no major safety signals reported.	[45] [133]
13 EGb 761 studies met inclusion; nine used in main meta‐analysis.	All studies used EGb761; daily doses 120–240 mg; 240 mg/day subgroup showed significant SKT benefit (SMD –0.71).	2381	Administration 12–52 weeks; five trials lasted 24 weeks.	Improved SKT scores in AD and vascular dementia	SKT SMDs favored EGb761 in all patients, combined AD+VaD, and AD alone; 240 mg/day effective in combined AD+VaD subgroup.	Total dropout rates “for any reason” did not differ between groups; dropout due to side effects was actually lower in Ginkgo groups (OR 1.72 [1.06, 2.80] favoring Ginkgo, i.e., fewer adverse‐event dropouts vs. placebo).	[134]
21 RCTs with 2608 patients were included.	Meta‐analysis compares *Ginkgo biloba* (alone or added to conventional medicine) versus placebo or conventional medicine alone; the MMSE/ADL effect sizes you quote are specifically for GB + conventional therapy versus conventional therapy alone in AD/MCI subgroups.	2608	Trials had variable durations; the MMSE and ADL estimates you quoted are for outcomes at 24 weeks in the relevant subgroups, not that all 21 trials were 24 weeks.	Correct for the combination therapy: At 24 weeks, GB + conventional medicine produced greater MMSE improvement in AD/MCI and better ADL scores in AD compared with conventional therapy alone.	At 24 weeks in AD, MMSE MD 2.39 (95% CI 1.28–3.50, *p* < 0.0001). There is also a separate MMSE estimate for MCI (MD 1.90, 95% CI 1.41–2.39, *p* < 0.00001). ADL MD −3.72 (95% CI −5.68 to −1.76, *p* = 0.0002).	Adverse events were mild and similar in frequency between Ginkgo and control; no significant difference in adverse reaction rates (*p* = 0.69).	[135]
Large, randomized, double‐blind, placebo‐controlled GuidAge trial.	Standardized *Ginkgo biloba* extract EGb 761, 120 mg twice daily.	2854 enrolled and randomized; 1406 received ≥ 1 dose EGb 761, 1414 placebo.	Participants were followed up for 5 years; enrollment 2002–2004 with 5‐year follow‐up.	HR 0.84, 95% CI 0.60–1.18; *p* = 0.306; “did not reduce the risk of progression to AD.”	This is exactly the reported hazard ratio and *p* value for probable AD at 5 years.	Incidence of adverse events, deaths, stroke, hemorrhagic and cardiovascular events did not differ between groups.	[136]

**Table 3:** Key clinical findings and results of *Huperzia serrata.*

Name	Population	Extract/dose	Duration	Main findings	Quantitative results	Safety profile	Ref.
Phase II trial of huperzine A in mild‐to‐moderate AD	Mild‐to‐moderate Alzheimer′s disease patients	Huperzine A, 200 *μ*g BID	16 weeks	Demonstrated safety and tolerability; no statistically significant cognitive benefit at 200 *μ*g BID compared to placebo.	Change in ADAS‐Cog at 16 weeks: 1.92‐point improvement (huperzine A) vs. 0.34‐point improvement (placebo); *p* = 0.07.	Demonstrated safety and tolerability.	[117]
Systematic review and meta‐analysis (20 RCTs)	1823 participants	Huperzine A	6–16 weeks (varied)	Beneficial effects on cognitive function and daily living activities; interpretation limited due to high risk of bias.	MMSE improved significantly at 8, 12, and 16 weeks; ADL favored huperzine A at 6, 12, and 16 weeks (effect sizes varied).	Reported as tolerated in included trials.	[118]
Meta‐analysis (8 AD trials and 2 VD trials)	Alzheimer′s disease and vascular dementia patients	Huperzine A	Variable; longer duration showed better efficacy	Significant improvements in MMSE and ADL scores in AD and VD patients.	Significant MMSE and ADL improvements; memory quotient improved.	Side effects mild‐to‐moderate.	[137]

A unique type of alkaloid called HupA may be useful in the treatment of AD. For those with the illness, it appears to improve cognitive abilities and simplify everyday tasks. It is generally safe and well‐tolerated, according to studies. When numerous studies are combined, there have been improvements in mental health and daily functioning, particularly when treatment is prolonged. These findings imply that its structure plays a crucial role in altering memory‐related brain activity and managing Alzheimer′s symptoms [117].

### 5.1. Comparative Clinical Evaluation of Major Chiral Natural Compounds in AD

Comparative analysis shows that the only chiral natural product with strong regulatory approval and reliable symptomatic efficacy is (−)‐galantamine. In large trials, GBE (EGb761) shows a moderate cognitive benefit but no preventive or disease‐modifying effect. Although Phase II data show dose‐dependent inconsistency, HupA exhibits promising stereoselective AChE inhibition. In small cohorts, saffron extracts show similar short‐term cognitive efficacy to conventional AChE inhibitors. On the other hand, ginsenosides, resveratrol, and EGCG show strong preclinical and mechanistic support but lack adequate large‐scale clinical validation. Overall, rather than having been shown to have disease‐modifying properties, the majority of chiral natural compounds currently offer symptomatic relief (Table 4).

**Table 4:** Comparative clinical and translational evaluation of major chiral natural compounds in AD.

Compound	Highest clinical evidence (since 2020)	Trial details (Phase, *n*)	Key quantitative outcome	Disease‐modifying evidence	Main limitation	Reference
(−)‐Galantamine	FDA‐approved; supported by multiple RCTs and high‐certainty meta‐analyses involving ~11,000 patients.	Most common trial duration is 24 weeks (6 months), with some studies up to 2 years.	Mean ADAS‐Cog improvement about –2.86 points vs placebo at 6 months, clinically meaningful.	No evidence of disease modification; purely symptomatic therapy without effect on amyloid or tau.	GI side effects; no plaque reduction evidence	[138] [139]
Huperzine A (natural 2R,9R,10R,11R)	Evidence mainly from small RCTs and some Phase II trials; meta‐analyses show mixed results.	Phase II trial of 16 weeks with 200 *μ*g BID dose; higher doses showed possible cognitive effects.	ADAS‐Cog change 1.92 vs 0.34 (placebo), p = 0.07 (NS); higher dose trend	ADAS‐Cog improvement trend (1.92 vs 0.34 placebo) at 16 weeks, but not statistically significant (p = 0.07).	Limited large‐scale, long‐term trials; inconsistent dose responses reported across studies.	[140] [86]
*Ginkgo biloba* (EGb 761)	Large RCT + multiple meta‐analyses	RCT (N = 2854, 5 years)	Prevention trial showed no significant risk reduction (HR = 0.84, p = 0.306); cognitive tests showed modest improvement with standardized mean differences around –0.7 to –0.9 for doses ≥120 mg/day.	No prevention effect	Heterogeneous extract composition	[141] [67]
Saffron (Crocin)	Small RCTs +2021 review	Four clinical trials	Saffron showed similar cognitive improvement to donepezil and memantine in short‐term trials (16–52 weeks).	No biomarker evidence	Small sample sizes	[142] [69]
(−)‐EGCG	Strong preclinical evidence and early‐phase human studies exist; no large phase III RCTs in Alzheimer′s disease.	Early trials; no large RCT	EGCG reduces amyloid‐beta aggregation and oxidative stress in preclinical models; human cognitive data are limited and inconclusive.	Mechanistic potential only	Poor bioavailability	[143]
Trans‐Resveratrol	Small double‐blind trial	12‐month RCT	Biomarker modulation; limited cognitive improvement	No plaque regression	Rapid metabolism	[144] [145]
Ginsenosides (Rg1/Rb1)	Early clinical + strong preclinical	Small trials	Cognitive improvement trends	No disease modification	Lack of Phase III trials	[146] [147]

## 6. Conclusion

AD results from complicated pathological processes such as A*β* aggregation, tau hyperphosphorylation, OS, and neuroinflammation. Present treatments mostly ease symptoms but do not stop the disease from getting worse. Natural products that have chiral phytoconstituents are attractive options since their stereochemistry affects how they interact with different biological targets. Chemicals such as (−)‐galantamine, HupA, EGCG, ginkgolides, crocin, and ginsenosides act on several targets, such as by inhibiting AChE/BChE, working against amyloid, acting as antioxidants, and protecting nerves. Of these, (−)‐galantamine is still the only chiral natural compound that is approved for clinical use, whereas the others have favorable preclinical and early clinical results. Structures such as chalcones, sulfonylhydrazones, and flavonoid derivatives also show how stereochemistry is important when creating multitarget anti‐Alzheimer agents. Still, there are problems like limited bioavailability, BBB penetration, and not enough large clinical studies. Future studies that focus on enantioselective characterization, better delivery methods, and well‐planned clinical trials will be needed to turn natural chiral structures into treatments that change the course of AD.

## Author Contributions

A.S.: conceptualization and drafting of the original manuscript; S.K.S.: manuscript writing and visualization; A.K.S.: reviewing and editing of the manuscript.

## Funding

No funding was received for this manuscript.

## Ethics Statement

The authors have nothing to report.

## Consent

The authors have nothing to report.

## Conflicts of Interest

The authors declare no conflicts of interest.

## Data Availability

No new dataset has been generated in this manuscript.
